# Identification of a genetic signature enriching for response to ibrutinib in relapsed/refractory follicular lymphoma in the DAWN phase 2 trial

**DOI:** 10.1002/cam4.4422

**Published:** 2021-11-17

**Authors:** Sriram Balasubramanian, Brendan Hodkinson, Stephen J. Schuster, Nathan H. Fowler, Judith Trotman, Georg Hess, Bruce D. Cheson, Michael Schaffer, Steven Sun, Sanjay Deshpande, Jessica Vermeulen, Gilles Salles, Ajay K. Gopal

**Affiliations:** ^1^ Janssen Research & Development Spring House Pennsylvania USA; ^2^ Lymphoma Program Abramson Cancer Center of the University of Pennsylvania Philadelphia Pennsylvania USA; ^3^ Department of Lymphoma/Myeloma The University of Texas MD Anderson Cancer Center Houston Texas USA; ^4^ Haematology Department, Concord Hospital University of Sydney Sydney New South Wales Australia; ^5^ Department of Hematology/Oncology Johannes Gutenberg‐University Mainz Germany; ^6^ Lombardi Comprehensive Cancer Center Georgetown University Hospital Washington District of Columbia USA; ^7^ Janssen Research & Development Raritan New Jersey USA; ^8^ Janssen Research & Development Leiden The Netherlands; ^9^ Hospices Civils de Lyon Université de Lyon Pierre‐Bénite Cedex, Lyon France; ^10^ Division of Medical Oncology Department of Medicine The University of Washington Seattle Washington USA; ^11^ Clinical Research Division Fred Hutchinson Cancer Research Center Lymphoma Program Seattle Cancer Care Alliance Seattle Washington USA

**Keywords:** biomarkers, genetic variants, lymphoma, mutations

## Abstract

**Background:**

The single‐arm DAWN trial (NCT01779791) of ibrutinib monotherapy in patients with relapsed/refractory follicular lymphoma (FL) showed an overall response rate (ORR) of 20.9% and a median response duration of 19.4 months. This biomarker analysis of the DAWN dataset sought to determine genetic classifiers for prediction of response to ibrutinib treatment.

**Methods:**

Whole exome sequencing was performed on baseline tumor samples. Potential germline variants were excluded; a custom set of 1216 cancer‐related genes was examined. Responder‐ versus nonresponder‐associated variants were identified using Fisher's exact test. Classifiers with increasing numbers of genes were created using a greedy algorithm that repeatedly selected genes, adding the most nonresponders to the existing “predicted nonresponders” set and were evaluated with 10‐fold cross‐validation.

**Results:**

Exome data were generated from 88 patient samples and 13,554 somatic mutation variants were inferred. Response data were available for 83 patients (17 responders, 66 nonresponders). Each sample showed 100 to >500 mutated genes, with greater variance across nonresponders. The overall variant pattern was consistent with previous FL studies; 75 genes had mutations in >10% of patients, including genes previously reported as associated with FL. Univariate analysis yielded responder‐associated genes *FANCA*, *HISTH1B*, *ANXA6*, *BTG1*, and *PARP10*, highlighting the importance of functions outside of B‐cell receptor signaling, including epigenetic processes, DNA damage repair, cell cycle/proliferation, and cell motility/invasiveness. While nonresponder‐associated genes included well‐known *TP53* and *CARD11*, genetic classifiers developed using nonresponder‐associated genes included *ATP6AP1*, *EP400*, *ARID1A*, *SOCS1*, and *TBL1XR1*, suggesting resistance to ibrutinib may be related to broad biological functions connected to epigenetic modification, telomere maintenance, and cancer‐associated signaling pathways (mTOR, JAK/STAT, NF‐κB).

**Conclusion:**

The results from univariate and genetic classifier analyses provide insights into genes associated with response or resistance to ibrutinib in FL and identify a classifier developed using nonresponder‐associated genes, which warrants further investigation.

**Trial registration**: NCT01779791.

## Graphical abstract

Using patient samples from the single‐arm DAWN trial (NCT01779791), we performed univariate and genetic classifier analyses to gain insights into genes associated with response (responders, *n* = 17) or lack of response (nonresponders, *n* = 66) to ibrutinib monotherapy in relapsed/refractory follicular lymphoma. Genetic classifiers were developed using nonresponder‐associated genes, and the top five genes associated with no response to ibrutinib included *ATP6AP1*, *EP400*, *ARID1A*, *SOCS1*, and *TBL1XR1*, suggesting that resistance to ibrutinib might be related to broad biological functions outside Bruton tyrosine kinase signaling.

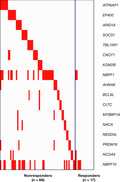

## 1 INTRODUCTION

Follicular lymphoma (FL) is the second most common histology of non‐Hodgkin lymphoma, with an incidence of approximately 5/100,000 in Western Europe and 3.4/100,000 (age‐adjusted) in the United States.^1^, ^2^ FL can be asymptomatic, which may not require immediate treatment, and patients with symptomatic FL typically receive chemoimmunotherapy (CIT) as the first‐line treatment.^1^, ^3^, ^4^ Despite the favorable survival outcomes with these therapies, FL is largely incurable, with approximately 20% of patients experiencing relapse within 24 months of initial therapy.^4^, ^5^ Several traditional therapies are associated with significant acute and delayed toxicity, especially in older or infirm patients. Early relapse and initial CIT resistance are robust predictors of inferior outcomes, with 5‐year survival rates of 34% to 50%,^4^ suggesting an area for treatment improvement.

Bruton tyrosine kinase (BTK) is a key component of the B‐cell receptor (BCR) signaling complex that plays an important role in the progression of B‐cell malignancies.^6^, ^7^ Ibrutinib is a first‐in‐class, oral, covalent inhibitor of BTK, which disrupts signaling pathways essential for malignant B‐cell adhesion, survival, and proliferation.^7^, ^8^ Because of its favorable efficacy and safety profile, as demonstrated in clinical trials,^9^, ^10^, ^11^, ^12^, ^13^, ^14^ ibrutinib has been approved for several B‐cell malignancies in the United States, European Union, and other countries,^8^, ^15^ and for chronic graft versus host disease in the United States.

In early‐phase clinical studies in relapsed FL, ibrutinib showed response rates ranging from 37.5% to 62.5%.^16^, ^17^, ^18^ In the phase 2 DAWN study (NCT01779791) in patients with relapsed/refractory FL who received ≥2 prior lines of therapy, ibrutinib monotherapy yielded an overall response rate (ORR) of 20.9%, with a 95% confidence interval spanning 13.7% to 29.7%, which did not meet the 18% lower‐bound threshold for the primary endpoint ORR.^19^ However, more than half (52.2%) of the responders achieved a complete response (CR), and the responses were durable, as demonstrated by a median response duration of 19.4 months. Preliminary biomarker analyses revealed that ibrutinib treatment decreased the level of regulatory T cells and increased T‐helper cell type 1–promoting cytokines in responders versus nonresponders, suggesting that T‐cell immunomodulatory effects may play a major role in the antitumor activity of ibrutinib in FL,^19^ but the role of tumor genetics was not examined.

Previous analyses have revealed that FL is a heterogeneous disease with varying genetic alterations underlying its pathobiology. The t(14;18)/*IGH*‐*BCL2* gene rearrangement resulting in *BCL2* overexpression is a hallmark of grade 1 to 2 FL, but is less common in grade 3 disease^20^, ^21^; in the latter, *BCL6* rearrangements (vs. *BCL2*) are often detected in t(14; 18).^21^ In addition, molecular genetic studies have identified recurrent somatic mutations that are significantly enriched in patients with FL.^20^, ^21^ These mutations affect genes in various signaling pathways potentially implicated in FL or lymphomagenesis, including epigenetic modifiers (*KMT2D*/*MLL2*, *CREBBP*, *EP300*), histone genes (*HIST1H1B*, *HIST1H1C*, *HIST1H1D*), vacuolar ATPase genes (*ATP6V1B2*, *VMA21*), and components of the BCR or CXCR4 signaling pathway (*CARD11*, *CXCR4*, *BTK*).^20^, ^21^ Notably, preliminary data from a small phase 2 study suggested that mutations in *CARD11*, which constitutively activates NF‐kB signaling downstream of BTK, may be associated with inferior response to ibrutinib in FL.^17^

Given the heterogeneity of FL and differential responses to treatment, identifying biomarkers that can potentially predict therapeutic benefit may improve clinical outcomes for specific subsets of patients. Here, we present the results of a biomarker analysis in which tumor samples from the DAWN study were examined to detect somatic mutations that could be used to identify patients with FL who are responsive to ibrutinib.

## 2 METHODS

### 2.1 Study design and patients

DAWN was a multicenter, open‐label, single‐arm, phase 2 study evaluating ibrutinib in relapsed/refractory FL. The study was conducted in accordance with International Conference on Harmonisation Good Clinical Practice guidelines and was approved by an independent institutional review board. All patients provided written informed consent. Detailed methodology for this trial is published elsewhere.^19^ In brief, ibrutinib 560 mg daily was administered until disease progression or unacceptable toxicity to patients aged 18 years or older who had a diagnosis of grade 1, 2, or 3a nontransformed FL, had been treated with at least two prior lines of therapy, and were relapsed/refractory to their last prior line of therapy with an anti‐CD20 monoclonal antibody−containing CIT regimen. The primary endpoint was ORR, including CR and partial response (PR), assessed by an independent review committee using the International Working Group Revised Response Criteria for Malignant Lymphoma.^22^

### 2.2 Whole exome sequencing

Formalin‐fixed, paraffin‐embedded (FFPE) tumor samples were collected at baseline for whole exome sequencing. Exome enrichment was performed using Nimblegen kits (Roche Sequencing Solutions), and sequencing libraries were created using KAPA construction kits (Roche Sequencing Solutions). Sequencing was performed using the HiSeq2500 platform (Illumina), achieving a mean target coverage of 60.7×.

### 2.3 Variant selection

Results of the sequencing analyses were visually examined by generating histograms illustrating variant allele frequency (VAF). This visual approach was used to qualitatively assess the degree to which somatic versus germline variants were present in the data by determining whether (i) low‐VAF variants were sufficiently represented in the results and (ii) variants with VAF values near 0.5 and 1.0 were sufficiently rare.

To improve the variant selection, an exome analysis pipeline was run on DNAnexus using raw FASTQ sequence data files (DNAnexus), and somatic variants were selected using multiple filters in the R software environment.^23^ Quality was assessed using FastQC 1.0.0, sequences were aligned to the hs37d5 genome build using the BWA‐MEM algorithm in BWA Software Package 0.5.9, alignments were recalibrated with the GATK 3.5 Exome Pipeline, and variants were annotated with MuTect 1.1.7, SnpEff 4.2 (using the GRCh37.75 database) and GEMINI 0.20.0 (modified by using non‐TCGA gnomAD and ExAC references).

Because the analysis was performed without matching normal samples, multiple filters were applied to nonsynonymous coding variants to rule out sequencing artifacts and germline variants. Detailed filtering criteria, imposed using base R functions on GEMINI outputs, are shown in Figure S1. All variants had a frequency of <0.001 in all four of the following databases of normal germline variants: ESP, 1kG, ExAC, and gnomAD. Potential somatic variants were further narrowed down for analysis to only those variants present in a selected set of 1216 known cancer‐related genes. Certain variants were marked as “deleterious” based on meta‐analytic support vector machine (MetaSVM) annotations in the database for nonsynonymous single nucleotide polymorphisms functional predictions (dbNSFP).

Variant frequencies were compared using Fisher's exact test to identify genes associated with responders (CR + PR) versus nonresponders (stable disease + progressive disease). Classifiers with increasing numbers of genes were developed using nonresponder‐associated genes, beginning with one gene and adding a single gene each time. Genes were added to classifiers based on their ranking by a greedy algorithm, which chose genes that allowed, in each iteration, the inference of the most additional nonresponders until all nonresponders were covered. During this process, ties were broken at random and a penalty was enforced for mutations in responders, such that the selected gene could add the most nonresponders after removing a proportion of patients equivalent to twice the proportion of responder patients with a mutation in the same gene. The performance of the classifiers was assessed by 10‐fold cross‐validation within the DAWN dataset, in which patients were binned as nonresponders if they harbored putative somatic mutations in any of the selected genes.

## 3 RESULTS

### 3.1 Variants summary

In total, 88 out of 110 patients enrolled in the DAWN study had FFPE tumor biopsy samples (one sample per patient) available for sequencing analyses. Sequencing generated 974,686 nonsynonymous coding variants and after filtering out potential errors and possible germline mutations, the variant count was 12,890. Following in‐house reprocessing and variant selection, the final VAF histogram showed a substantially increased somatic versus germline ratio for the variants (Figure S2). The VAF values across multiple patients for *EZH2*‐*Tyr646* and *STAT6*‐*Asp419*, two known somatic FL‐associated mutations,^24^, ^25^ all fell below 0.4, indicating filters were appropriately applied.

**FIGURE 1:**
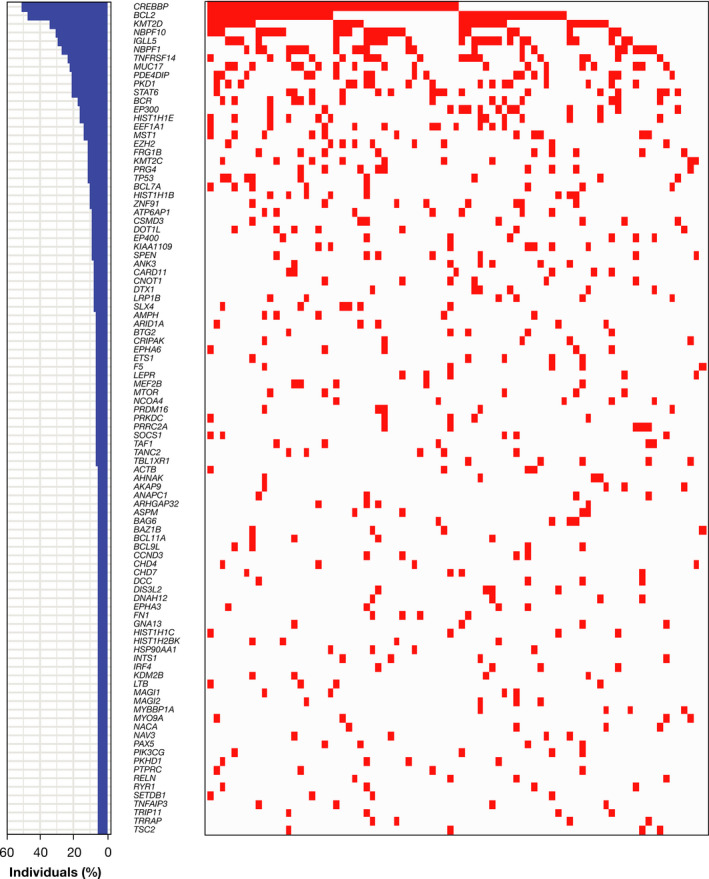
Cancer‐associated genes of interest that are mutated in more than three patients with follicular lymphoma. Left panel shows the percentage of individuals with a mutation in each gene; right panel shows the distribution of mutations in those genes in the 83 patients with response/nonresponse data

Of 88 patients with exome data, 83 had response data per independent review committee assessment (17 responders and 66 nonresponders) and were included in this analysis. The background demographic and disease characteristics summarized in Table S1 were generally similar to those published for the primary study population.^19^ The number of mutated genes in each sample varied from 100 to ≥500. A larger number of samples from nonresponders than responders led to greater genetic variance across nonresponder samples (Figure S3). Genes of interest from the selected cancer‐related gene set, restricted to gene mutations occurring in >3 patients, revealed mutations in several genes previously reported in FL (e.g., *CREBBP*, *BCL2*, *KMT2D*; Figure 1). The full heatmap of genes mutated in >10% of samples (75 genes) in the 83 patients with response data is presented in Figure S4.

Univariate analysis of variants from the selected genes in responders versus nonresponders is summarized in Table 1. As the number of responders was small, relatively few variants in any given gene could result in a large proportion of responders sharing that mutated gene. As a result, univariate analysis largely yielded genes mutated significantly more frequently in ibrutinib responders, although these genes were still found to have very few variants overall, such as *FANCA*, *HISTH1B*, *ANXA6*, *BTG1*, *DIAPH1*, *PARP10*, *PBRM1*, *PRDM1*, *RAD50*, and *RECQL4* (Table 1). Several selected gene mutations more frequent in nonresponders, but not significantly associated with the nonresponder status (*p* value ≤0.05), are summarized in Table 2.

**TABLE 1:** Univariate analysis of gene variants more frequently mutated in responders from the selected cancer‐related gene set*

Gene mutation	Responder (*n* = 17) *n* (%)	Nonresponder (*n* = 66) *n* (%)	Odds ratio (95% CI)	*p* value
*FANCA*	3 (17.6)	0 (0.0)	Inf (1.721‐Inf)	0.007
*HIST1H1B*	5 (29.4)	3 (4.5)	8.417 (1.426–61.654)	0.008
*ANXA6*	2 (11.8)	0 (0.0)	Inf (0.750‐Inf)	0.040
*BTG1*	2 (11.8)	0 (0.0)	Inf (0.750‐Inf)	0.040
*DIAPH1*	2 (11.8)	0 (0.0)	Inf (0.750‐Inf)	0.040
*PARP10*	2 (11.8)	0 (0.0)	Inf (0.750‐Inf)	0.040
*PBRM1*	2 (11.8)	0 (0.0)	Inf (0.750‐Inf)	0.040
*PRDM1*	2 (11.8)	0 (0.0)	Inf (0.750‐Inf)	0.040
*RAD50*	2 (11.8)	0 (0.0)	Inf (0.750‐Inf)	0.040
*RECQL4*	2 (11.8)	0 (0.0)	Inf (0.750‐Inf)	0.040
*TANC2*	3 (17.6)	2 (3.0)	6.634 (0.693–86.599)	0.056
*KMT2C*	4 (23.5)	5 (7.6)	3.677 (0.640–19.847)	0.080
*LRP1B*	3 (17.6)	3 (4.5)	4.391 (0.532–36.391)	0.097
*MAST2*	2 (11.8)	1 (1.5)	8.352 (0.410–516.902)	0.105
*MYCBP2*	2 (11.8)	1 (1.5)	8.352 (0.410–516.902)	0.105
*NDRG1*	2 (11.8)	1 (1.5)	8.352 (0.410–516.902)	0.105
*NEK1*	2 (11.8)	1 (1.5)	8.352 (0.410–516.902)	0.105
*SETD2*	2 (11.8)	1 (1.5)	8.352 (0.410–516.902)	0.105
*SMARCA4*	2 (11.8)	1 (1.5)	8.352 (0.410–516.902)	0.105
*BAG6*	2 (11.8)	2 (3.0)	4.167 (0.281–61.877)	0.184
*CHD4*	2 (11.8)	2 (3.0)	4.167 (0.281–61.877)	0.184
*NAV3*	2 (11.8)	2 (3.0)	4.167 (0.281–61.877)	0.184
*TNFAIP3*	2 (11.8)	2 (3.0)	4.167 (0.281–61.877)	0.184

Abbreviations: CI, confidence interval; Inf, infinite.

* Results are shown only for genes with *p* values <0.2.

**TABLE 2:** Selected genes from the univariate analysis more frequently mutated in nonresponders

Gene mutation	Responder (*n* = 17) *n* (%)	Nonresponder (*n* = 66) *n* (%)	Odds ratio (95% CI)	*p* value
*NBPF1*	2 (11.8)	20 (30.3)	0.310 (0.032–1.539)	0.216
*ATP6AP1*	0 (0.0)	7 (10.6)	0.000 (0.000–2.689)	0.335
*EP400*	0 (0.0)	7 (10.6)	0.000 (0.000–2.689)	0.335
*CNOT1*	0 (0.0)	6 (9.1)	0.000 (0.000–3.327)	0.338
*DTX1*	0 (0.0)	6 (9.1)	0.000 (0.000–3.327)	0.338
*SLX4*	0 (0.0)	6 (9.1)	0.000 (0.000–3.327)	0.338
*MUC17*	2 (11.8)	16 (24.2)	0.420 (0.042–2.135)	0.340
*MST1*	1 (5.9)	10 (15.2)	0.353 (0.008–2.841)	0.446
*PKD1*	2 (11.8)	15 (22.7)	0.457 (0.046–2.340)	0.503
*NBPF10*	4 (23.5)	21 (31.8)	0.662 (0.140–2.497)	0.570
*ARID1A*	0 (0.0)	5 (7.6)	0.000 (0.000–4.310)	0.578
*BTG2*	0 (0.0)	5 (7.6)	0.000 (0.000, 4.310)	0.578
*ETS1*	0 (0.0)	5 (7.6)	0.000 (0.000–4.310)	0.578
*F5*	0 (0.0)	5 (7.6)	0.000 (0.000–4.310)	0.578
*PRDM16*	0 (0.0)	5 (7.6)	0.000 (0.000–4.310)	0.578
*PRKDC*	0 (0.0)	5 (7.6)	0.000 (0.000–4.310)	0.578
*PRRC2A*	0 (0.0)	5 (7.6)	0.000 (0.000–4.310)	0.578
*SOCS1*	0 (0.0)	5 (7.6)	0.000 (0.000–4.310)	0.578
*TAF1*	0 (0.0)	5 (7.6)	0.000 (0.000–4.310)	0.578
*TBL1XR1*	0 (0.0)	5 (7.6)	0.000 (0.000–4.310)	0.578
*FRG1B*	1 (5.9)	8 (12.1)	0.457 (0.010–3.858)	0.678
*PRG4*	1 (5.9)	8 (12.1)	0.457 (0.010–3.858)	0.678
*TP53*	1 (5.9)	8 (12.1)	0.457 (0.010–3.858)	0.678
*BCL7A*	1 (5.9)	7 (10.6)	0.530 (0.011–4.647)	1.000
*ZNF91*	1 (5.9)	7 (10.6)	0.530 (0.011–4.647)	1.000
*CARD11*	1 (5.9)	5 (7.6)	0.765 (0.015–7.571)	1.000

Abbreviation: CI, confidence interval.

To determine whether gene mutations that were more frequent in responders were enriched in subgroups of patients who achieved a CR versus a PR, we compared the frequencies of mutations in patients who achieved a CR (*n* = 9) with a non‐CR group, including patients with a PR (*n* = 8), as well as all nonresponders (*n* = 66; total 74 patients). Gene mutations significantly enriched in patients with a CR (vs. non‐CR) are presented in Table S2. Generally, with a caveat of small sample size, the results indicate that the majority of mutations significantly enriched in responders (*HISTH1B*, *ANXA6*, *BTG1*, *PARP10*, *PBRM1*; Table 1) are also associated with a CR, except *FANCA* mutation, which was found in two patients with a PR and in one patient with a CR. A direct comparison between responders with a CR versus those with a PR was inconclusive because of small sample size (9 vs. 8; Table S3). Of note, *BCL2* mutation was enriched in patients with CR versus PR (6 vs. 2), while *DIAPH1*, *PKD1*, and *TNFAIP3* mutations occurred only in patients who achieved a PR (two each). Mutations in several genes, including *ANXA6*, *BTG1*, and *PARP10*, were found only in patients with CR (two each), but none of these results reached statistical significance (Table S3).

### 3.2 Classifier development and cross‐validation

**FIGURE 2:**
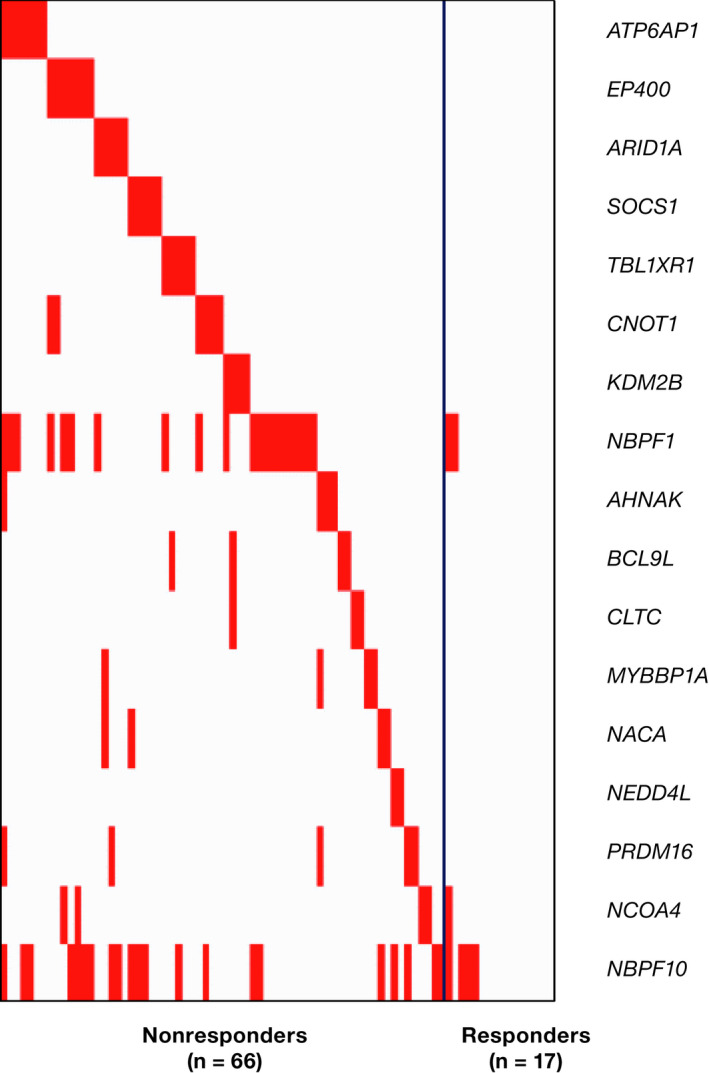
Heatmap of ranked nonresponder gene mutations in ibrutinib‐treated patients with follicular lymphoma

**FIGURE 3:**
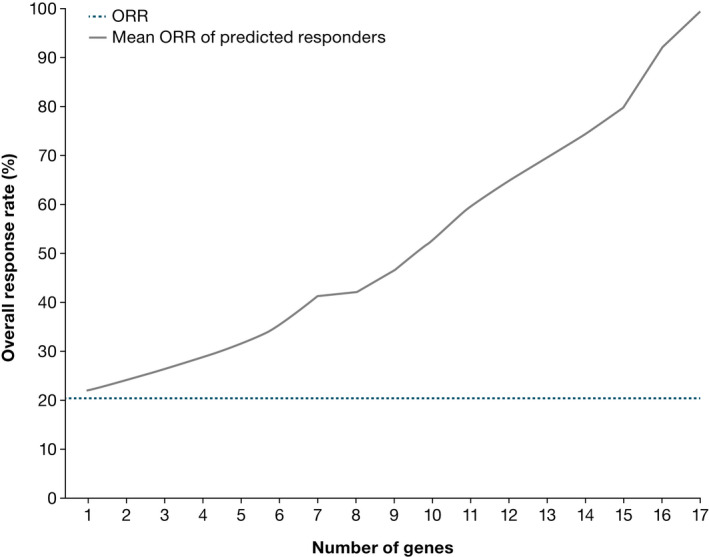
Mean overall response rate (ORR) of predicted responders (gray line) based on 10‐fold cross‐validation for different responder/nonresponder classification models containing an increasing number of genes. The dotted blue line represents the ORR of the entire patient cohort regardless of classification. ORR, overall response rate

Given that genes that were exclusively mutated in responders all had modest numbers of patients supporting them as biomarkers, genes that were mutated more often in nonresponders (vs. responders) were targeted for classifier development. Development started with a single gene that was ranked most informative, and subsequent classifiers were created by adding, one at a time, another gene in the order of decreasing new information. For the selected panel, 17 classifier models were developed including variants in *ATP6AP1*, *EP400*, *ARID1A*, *SOCS1*, *TBL1XR1*, *CNOT1*, and *KDM2B* (Figure 2). In 10‐fold cross‐validation, performance, indicated by the ORR of the predicted responder group, increased steadily as more genes were added (Figure 3). Each tested classifier produced a moderate increase in response rate.

### 3.3 Genes of interest in nonresponders

**FIGURE 4:**
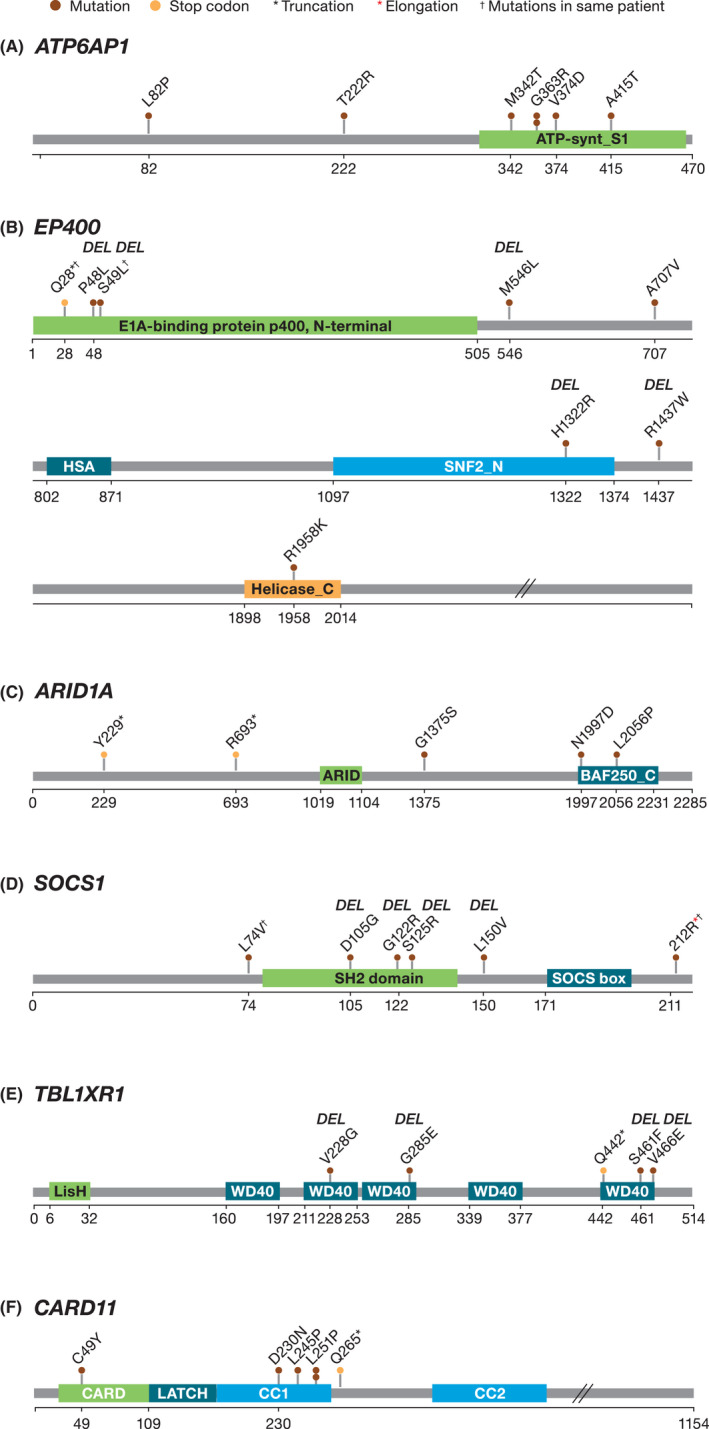
Plots of somatic mutations in the genes of interest associated with the lack of response to ibrutinib: (A) *ATP6AP1*, (B) *EP400*, (C) *ARID1A*, (D) *SOCS1*, (E) *TBL1XR1*, and (F) *CARD 11*. DEL = the mutation was predicted to be deleterious by metaSVM

Among the 83 patients in the DAWN study with both exome data and responder/nonresponder status, the top five mutations in the gene classifier, which were also exclusively found in nonresponders, included *ATP6AP1*, *EP400*, *ARID1A*, *SOCS1*, and *TBL1XR1* (Figure 4).

Six different mutations in seven patients were identified in the *ATP6AP1* gene. Five of these patients had mutations in the ATP‐synthase S1 region in the C‐terminal end, two of whom had the same *G363R* mutation in this region (Figure 4A). Seven patients had eight somatic mutations in the *EP400* gene, five of whom had mutations marked as “deleterious” by metaSVM, including one patient with two deleterious mutations in the N‐terminal region (Figure 4B). Five patients had five different mutations across the length of the *ARID1A* gene; two of these mutations were located in the BAF250 C domain and two other introduced premature stop codons (Figure 4C). Five patients had six mutations in the *SOCS1* gene; five of these were located in the SH2 domain and were predicted as deleterious by metaSVM (Figure 4D). Five patients had five mutations in the *TBL1XR1* gene, of which four were predicted as deleterious by metaSVM; the remaining one mutation introduced a premature stop codon, which would likely impair the function of the protein (Figure 4E).

### 3.4 *CARD11* and *TP53* analyses

Six of 83 patients in the DAWN study with both exome data and response data had inferred somatic *CARD11* mutations (C49Y, D230N [also found in the Bartlett et al. study^17^], L245P, L251P [in two different patients], and Q265). In our analysis, five out of six patients with *CARD11* mutations were nonresponders; four of these patients had *CARD11* mutations in the coiled‐coil domain and one patient had *CARD11* variant with a premature stop codon (Figure 4F). Interestingly, the remaining six patients were a responder, with C49Y mutation in the CARD domain (Figure 4F). Nine of 83 patients had mutations in the *TP53* gene, and eight of these patients were nonresponders. The presence of one responder in each group of patients with *CARD11* and *TP53* mutations precluded inclusion of these genes in the genetic classifier described above.

## 4 DISCUSSION

By using whole exome sequencing and a custom variant selection scheme, this analysis identified somatic gene mutations potentially associated with response/nonresponse to ibrutinib in patients with relapsed/refractory FL. Comparison to previous genetic studies in FL established the validity of the variant selection scheme developed in this study, as the overall pattern of variant frequencies identified here was generally comparable to the earlier studies,^17^, ^20^, ^26^, ^27^ with a few exceptions. In a sequencing‐based biomarker study in FL (*n* = 105),^20^ the rates of *KMT2D* and *TNFRSF14* mutations were higher than those seen in this analysis, but a large proportion of the mutations was represented by indels, which were not assessed in the current study and explains the discrepancy. In addition, the rates of *BCL2* and *MUC4* mutations were higher in the present analysis, but the previous study showed a higher number of somatic mutations called in these genes when a matched normal sample was not present. Taking these factors into account, the overall results of the two studies compare favorably.

In the univariate analysis presented herein, many responder‐associated gene mutations have shown functions outside of BCR signaling. Mutations in histone genes and epigenetic modifiers have been frequently reported in studies of FL,^20^, ^21^, ^26^ suggesting that epigenetic dysregulation is a major mechanism driving the pathogenesis of FL. In this study, the occurrence of some of these mutations in responders to ibrutinib may suggest a link to an ibrutinib‐related mechanism of action. *HIST1H1B* is a histone H1 gene that was significantly mutated in patients with FL, with all known alterations being missense mutations.^20^ *PBRM1*, a gene also found mutated in FL, is a subunit of the chromatin‐remodeling SWI/SNF complexes.^20^, ^28^ *PARP10* has a role in the regulation of chromatin and gene transcription and cell proliferation,^29^ and *PARP10* knockdown results in genomic instability and DNA damage hypersensitivity.^30^ Two other frequently mutated genes in responders, *FANCA* and *RAD50*, with three and two mutations each, respectively, are associated with DNA damage repair, and mutations in these genes are known to sensitize tumors to chemotherapeutics.^31^, ^32^, ^33^ The *BTG1* gene, mutated exclusively in two responders with a CR in this analysis, was previously characterized as a negative regulator of cell cycle progression and cell proliferation,^34^ and mutations in this gene were enriched in human B‐cell precursor acute lymphoblastic leukemia and were associated with inferior outcomes.^35^ Lastly, protein expression of the *ANXA6* gene (mutated only in two patients with a CR) is required for membrane localization of activated EGFR and persistent activation of MAP kinaseERK1/2 and PI3K/Akt pathways in invasive breast cancer cells. Depletion of ANXA6 expression in these cells leads to degradation of activated EGFR, inhibition of cell motility and invasiveness, and increased sensitivity to the EGFR‐targeted tyrosine kinase inhibitors.^36^ Taken together, these data suggest that the biology relevant to ibrutinib activity in FL may extend beyond the BTK‐NF‐κB pathway, to epigenetic changes in the expression of key tumor‐related genes, gene and protein regulation, DNA repair, cell cycle progression, and other cellular processes. However, the fact that relatively few conserved gene mutations were identified in responders, mostly due to the modest numbers of samples, represents a limitation of the univariate analysis and obscures the interpretation of the link between gene mutations and response to ibrutinib.

The univariate analysis did not identify any significant nonresponder‐associated gene mutations, which otherwise would be of special interest as they may activate survival mechanisms that bypass BTK, including the mTOR and JAK/STAT pathways, and confer resistance to ibrutinib.^37^, ^38^ This include genes previously linked with poor prognosis in FL, such as *TP53* and *CARD11,* ^39^ which did not reach significance in the univariate analysis described herein. When no predictive value of a single mutation can be ascertained, the classifier integration may become predictive. Further investigation of the top five ranked nonresponder‐associated genes from gene classifier models (*ATP6AP1*, *EP400*, *ARID1A*, *SOCS1*, and *TBL1XR1*) has suggested potential mechanisms underlying resistance to ibrutinib. In *ATP6AP1*, a v‐ATPase complex mediating mTORC1 activation,^37^ the majority of mutations were in the ATP‐synthase S1 region in the C‐terminal end, which is hypothesized to convey a “false” amino acid sufficiency signal or alter interactions between v‐ATPase and downstream signaling molecules, resulting in aberrant mTORC1 activation.^37^ As Akt/mTOR signaling is downstream of BTK, increased mTOR activity may reduce the effectiveness of ibrutinib.^40^ Mutations in EP400, a chromatin‐remodeling protein and a transcriptional repressor, may activate gene expression implicated in FL oncogenesis, as is the case with other cancers.^41^ *ARID1A* mutations have been reported in many types of human cancers,^42^ including FL.^26^ In the DAWN dataset, mutations in *ARID1A* showed a distribution pattern consistent with a previous analysis in FL.^26^ Of these mutations, one (R693*) was previously reported in FL,^26^ and two introduced premature stop codons in the C terminal. Given that *ARID1A* is a negative regulator of TERS, loss of its expression caused by inactivating mutations would enhance TERT transcription, conferring a survival advantage for tumor cells by maintaining their telomeres.^42^ *SOCS1* is a negative regular of JAK/STAT signaling. Mutations in *SOCS1*, including those in the SH2 domain, were reported in B‐cell malignancies such as diffuse large B‐cell lymphoma and FL.^27^ As the SH2 domain mediates the binding of *SOCS1* to JAK, leading to the subsequent inactivation of the JAK/STAT signaling, mutations in this domain detected in our analysis may prolong the activation of the JAK/STAT pathway.^38^ In chronic lymphocytic leukemia, preliminary data have shown that activated JAK/STAT signaling potentially contributed to the resistance to ibrutinib.^43^ *TBL1XR1*, a transducin β‐like 1 X‐linked receptor 1, is a tumor‐suppressor gene with E3 ubiquitin ligase activity, mediating transcriptional repression through NF‐κB and WNT signaling pathways.^44^ Oncogenic NF‐κB‐activating mutations were found in acute lymphoblastic leukemia, splenic marginal zone lymphoma, and primary central nervous system lymphoma.^44^ In the DAWN study, five *TBL1XR1* somatic mutations were found in nonresponders, indicating that mutations in this gene lead to alternative, BTK‐independent NF‐κB‐activation and, therefore, resistance to ibrutinib, as reported in certain MCL cell lines.^45^

In a previous phase 2 consortium study of ibrutinib in FL, *CARD11* was identified as the primary “nonresponder” gene, with eight mutations reported in five patients (*n* = 31), all of whom were nonresponders.^17^ In our analysis, this trend was generally confirmed by identifying *CARD11* mutations in six patients, five of whom were nonresponders (mutations were mostly in the coiled‐coil domain). One *CARD11* mutation (C49Y, in the CARD domain) was found in a responder who achieved a PR after 5.5 months of ibrutinib treatment. This patient was a 53‐year‐old white female with stage II FL and low‐risk Follicular Lymphoma International Prognostic Index score. The C49Y *CARD11* variant has been reported in diffuse large B‐cell lymphoma and was identified in an unbiased screen for gain‐of‐function *CARD11* mutants capable of activating NF‐κB and promoting human diffuse large B‐cell lymphoma tumor growth in vitro.^46^ This variant has also been reported to predict a gain of function resulting in B‐cell expansion with NF‐κB and T‐cell anergy (BENTA) disease.^47^ However, some patients with C49Y *CARD11* germline mutations present with relatively mild BENTA, suggesting that the gain‐of‐function effect of a C49Y *CARD11* variant is weaker than that of a mutation in the coiled‐coil or LATCH domain, making patient outcome highly context dependent.^48^ It is possible that the responder in our study might have had a germline mutation in the CARD domain that was associated with a weak *CARD11* activation, but nonetheless responded to ibrutinib due to other factors, such as relatively young age and overall low‐risk disease.

In summary, the genes found to be most useful for predicting lack of response to ibrutinib in the DAWN dataset demonstrate a variety of biological functions. Some of these genes act directly through the BTK‐NF‐κB pathway. Among the top‐ranked nonresponder genes, *ATP6AP1* mutations may overcome BTK inhibition by activating mTOR signaling,^37^, ^40^ and mutated *TBL1XR1* activates NF‐κB, which could trigger resistance to ibrutinib treatment.^44^, ^45^ The nonresponder‐associated gene *SOCS1*, a regulator of the JAK/STAT pathway, acts not only as an intracellular pathway component, but also as a modulator of the tumor microenvironment.^38^ *CARD11*, which is associated with lack of response to ibrutinib,^17^ encodes a scaffold protein required for BCR activation of NF‐κB signaling and implicated in lymphoma tumorigenesis,^46^ indicating that resistance to ibrutinib may be more closely linked to NF‐kB‐related pathways in the tumor. Interestingly, most genes in the final classifier models, except *ATP6AP1*, have not been previously reported as major drivers of FL, and somatic mutations in the main genes known to be associated with FL did not appear to effectively predict the response to ibrutinib. These observations may have revealed additional molecular mechanisms contributing to FL pathogenesis or unique aspects of ibrutinib's activity in FL patients, which warrants further investigation.

The ORR with ibrutinib monotherapy in relapsed/refractory FL ranged from 20.9% in the DAWN study to 62.5% in the long‐term follow‐up phase 1 trial, with wide variation potentially attributed to different response assessment approaches, patient populations, and follow‐up periods.^16^, ^17^, ^18^ The identification of nonresponder‐associated genes has contributed to the understanding of possible molecular mechanisms underlying ibrutinib resistance, including the activation of mTOR and JAK/STAT signaling and TERT‐mediated telomere maintenance. These results may lay the groundwork for further development of ibrutinib combination therapy with a partner that overcomes resistance to ibrutinib and improves outcomes in FL.

The main limitations of this study include an unbalanced analysis cohort with more nonresponders than responders; the absence of normal matching samples; the classifier gene selection strategy may have resulted in “overfitting” of the model; and the lack of an independent cohort for external validation.

In conclusion, this analysis developed a viable approach to identify somatic mutations associated with response to ibrutinib in FL. Our results suggest that the pattern of response to ibrutinib in FL may be linked to a variety of non‐BTK‐related pathways and microenvironmental interactions. The genetic data suggest that resistance to ibrutinib in FL may be related to epigenetic modification and telomere maintenance, in addition to cancer‐associated signaling pathways (mTOR, JAK/STAT, NF‐κB). However, these results need to be validated in additional patient datasets/prospective studies to fully determine their clinical predictive value.

## CONFLICT OF INTEREST

**SB:** Employment: Janssen Research & Development; Equity ownership: Janssen Research & Development. **BH, MS, SS, SD, and JV:** Employment: Janssen Research & Development. **SJS**: Consultancy and research funding: Novartis, Pharmacyclics, Celgene; Research funding: Gilead, Janssen Research & Development, Hoffmann‐La Roche, Merck; Board of directors or advisory committees: Nordic Nanovector; Consultancy: Genentech, Acerta. **NHF:** Consulting or advisory role: Pharmacyclics, Janssen; Research funding: Pharmacyclics, Janssen. **JT:** Research funding to institution: Janssen, PCYC, Roche, BeiGene, Takeda, Celgene; Cooperative group funding: Janssen; Advisory role (unremunerated): Janssen, Roche, Celgene, Takeda. **GH**: Research support: Roche, Pfizer, Celgene, CTI; Consultant: Roche, Pfizer, Janssen, Celgene, AbbVie; Honoraria: Roche, Pfizer, Janssen, Celgene, AbbVie; Advisory board: Roche, Pfizer, Janssen, Celgene, AbbVie. **BDC:** Advisory board: Acerta, AbbVie, Roche‐Genentech, Celgene, TG Therapeutics; Research funding to institution: Gilead, Pharmacyclics, Acerta, TG Therapeutics, AbbVie. **GS**: Research funding: Roche, Celgene; Consultancy: Novartis; Honoraria: AbbVie, Acerta, Amgen, Celgene, Epizyme, Gilead, Janssen, Merck, Morphosys, Novartis, Pfizer, Roche, Servier, Takeda, Celgene, Mundipharma. **AKG:** Research funding: Merck, Janssen, Spectrum, Takeda, Bristol Myers Squibb, Pfizer, Seattle Genetics, Gilead; Donations: Frank and Betty Vandermeer; Consulting fees: Seattle Genetics, Gilead, Janssen, Brim, Aptevo, Genzyme.

## ETHICAL APPROVAL

This secondary analysis did not require ethical approval. The primary phase 2 study DAWN (NCT01779791) was conducted in accordance with International Conference on Harmonisation Good Clinical Practice guidelines and was approved by an independent institutional review board. All patients provided written informed consent (Gopal AK, Schuster SJ, Fowler NH, et al. Ibrutinib as treatment for patients with relapsed/refractory follicular lymphoma: results from the open‐label, multicenter, phase II DAWN study. *J Clin Oncol*. 2018;36(23):2405–2412.)

## Supporting information

Supplementary Material

## Data Availability

The data sharing policy of the Janssen Pharmaceutical Companies of Johnson & Johnson is available at www.janssen.com/clinical‐trials/transparency. Request for access to data from select studies can be submitted through the Yale Open Data Access (YODA) Project site at yoda.yale.edu.
