# Supplementary material for: Identification of a genetic signature enriching for response to ibrutinib in relapsed/refractory follicular lymphoma in the DAWN phase 2 trial
Source: Cancer Med. 2021 Nov 17;11(1):61–73. doi: 10.1002/cam4.4422 (PMC8704158; doi:10.1002/cam4.4422)

**SUPPORTING INFORMATION**

**Table S1.** Baseline demographic and disease characteristics for patients with exome and response data

|  | **Biomarker cohort treated with ibrutinib**  **N = 83** | |
| --- | --- | --- |
| Age, median (range), years | | 61.0 (28-87) |
| Male, n (%) | | 53 (63.9) |
| ECOG performance status, n (%) | | |
| 0 | | 45 (54.2) |
| 1 | | 38 (45.8) |
| FL stage, n (%) | | |
| I | | 1 (1.2) |
| II | | 10 (12.0) |
| III | | 28 (33.7) |
| IV | | 44 (53.0) |
| FLIPI score, n (%) | | |
| 0-1 | | 16 (19.3) |
| 2 | | 20 (24.1) |
| 3-5 | | 47 (56.6) |
| Tumor bulk ≤ 6 cm, n (%) | | 71 (85.5) |
| LDH > ULN, n (%) | | 34 (41.0) |
| Median (range) prior LOT | | 3.0 (2-13) |
| Refractory/relapsed* to the last prior LOT, n (%) | | |
| Relapsed | | 48 (57.8) |
| Refractory | | 35 (42.2) |

Abbreviations: ECOG, Eastern Cooperative Oncology Group; FL, follicular lymphoma; FLIPI, Follicular Lymphoma International Prognostic Index; LDH, lactate dehydrogenase; LOT, lines of therapy.

*Relapsed disease was defined as relapsed or disease progression after achieving at least partial response to the last regimen prior to study entry. Refractory disease was defined as failure to achieve at least partial response to the last regimen prior to study entry.

**Table S2****.** Univariate analysis of gene variants more frequently mutated in patients who achieved a CR from the selected cancer-related gene set*: a comparison versus a non-CR group^†^

| **Gene mutation** | **CR (n = 9)** | **non-CR (n = 74)** | **Odds ratio (95% CI)** | ***P* value** |
| --- | --- | --- | --- | --- |
|  | n (%) | n (%) |  |  |
| *HIST1H1B* | 4 (44.4) | 4 (5.4) | 13.116 (1.876-97.502) | 0.004 |
| *TANC2* | 3 (33.3) | 2 (2.7) | 16.664 (1.600-235.891) | 0.008 |
| *ANXA6* | 2 (22.2) | 0 (0.0) | Inf (1.644-Inf) | 0.011 |
| *BTG1* | 2 (22.2) | 0 (0.0) | Inf (1.644-Inf) | 0.011 |
| *PARP10* | 2 (22.2) | 0 (0.0) | Inf (1.644-Inf) | 0.011 |
| *LRP1B* | 3 (33.3) | 3 (4.1) | 11.142 (1.230-103.526) | 0.015 |
| *MYCBP2* | 2 (22.2) | 1 (1.4) | 19.227 (0.901-1233.367) | 0.030 |
| *NEK1* | 2 (22.2) | 1 (1.4) | 19.227 (0.901-1233.367) | 0.030 |
| *AMPH* | 2 (22.2) | 3 (4.1) | 6.489 (0.469-67.556) | 0.089 |
| *EP300* | 3 (33.3) | 10 (13.5) | 3.141 (0.439-17.882) | 0.144 |
| *CREBBP* | 7 (77.8) | 35 (47.3) | 3.841 (0.671-40.302) | 0.156 |
| *KIAA1109* | 2 (22.2) | 5 (6.8) | 3.846 (0.312-29.796) | 0.165 |
| *PKD1* | 0 (0.0) | 17 (23.0) | 0.000 (0.000-1.916) | 0.193 |
| *EZH2* | 2 (22.2) | 7 (9.5) | 2.691 (0.230-18.604) | 0.251 |
| *KMT2C* | 2 (22.2) | 7 (9.5) | 2.691 (0.230-18.604) | 0.251 |
| *BCL2* | 6 (66.7) | 33 (44.6) | 2.458 (0.481-16.344) | 0.294 |
| *FANCA* | 1 (11.1) | 2 (2.7) | 4.368 (0.068-93.256) | 0.294 |
| *NDRG1* | 1 (11.1) | 2 (2.7) | 4.368 (0.068-93.256) | 0.294 |
| *SETD2* | 1 (11.1) | 2 (2.7) | 4.368 (0.068-93.256) | 0.294 |
| *SMARCA4* | 1 (11.1) | 2 (2.7) | 4.368 (0.068-93.256) | 0.294 |
| *BAG6* | 1 (11.1) | 3 (4.1) | 2.903 (0.050-41.567) | 0.374 |
| *CARD11* | 0 (0.0) | 6 (8.1) | 0.000 (0.000-7.581) | 1 |
| *DIAPH1* | 0 (0.0) | 2 (2.7) | 0.000 (0.000-45.612) | 1 |

Abbreviations: CI, confidence interval; CR, complete response; Inf, infinity

*Top 10 genes with lowest *P* values and selected genes of interest from Table 1 are shown.

^†^Non-CR group includes patients who achieved partial response (n = 8) and all nonresponders (n = 66).

**Table S3.** Univariate analysis comparing gene variants in patients with a CR versus those with a PR, from the selected cancer-related gene set*

| **Gene mutation** | **CR (n = 9)**  n (%) | **PR (n = 8)**  n (%) | **Odds ratio (95% CI)** | ***P* value** |
| --- | --- | --- | --- | --- |
| *BCL2* | 6 (66.7) | 2 (25.0) | 5.327 (0.515-87.727) | 0.153 |
| *LRP1B* | 3 (33.3) | 0 (0.0) | Inf (0.394-Inf) | 0.206 |
| *TANC2* | 3 (33.3) | 0 (0.0) | Inf (0.394-Inf) | 0.206 |
| *TNFRSF14* | 3 (33.3) | 0 (0.0) | Inf (0.394-Inf) | 0.206 |
| *DIAPH1* | 0 (0.0) | 2 (25.0) | 0.000 (0.000-4.598) | 0.206 |
| *PKD1* | 0 (0.0) | 2 (25.0) | 0.000 (0.000-4.598) | 0.206 |
| *TNFAIP3* | 0 (0.0) | 2 (25.0) | 0.000 (0.000-4.598) | 0.206 |
| *HIST1H1B* | 4 (44.4) | 1 (12.5) | 5.059 (0.352-313.617) | 0.294 |
| *KMT2D* | 2 (22.2) | 4 (50.0) | 0.309 (0.019-3.356) | 0.335 |
| *CREBBP* | 7 (77.8) | 4 (50.0) | 3.237 (0.298-51.965) | 0.335 |
| *AMPH* | 2 (22.2) | 0 (0.0) | Inf (0.169-Inf) | 0.471 |
| *ANXA6* | 2 (22.2) | 0 (0.0) | Inf (0.169-Inf) | 0.471 |
| *BTG1* | 2 (22.2) | 0 (0.0) | Inf (0.169-Inf) | 0.471 |
| *EZH2* | 2 (22.2) | 0 (0.0) | Inf (0.169-Inf) | 0.471 |
| *KIAA1109* | 2 (22.2) | 0 (0.0) | Inf (0.169-Inf) | 0.471 |
| *MYCBP2* | 2 (22.2) | 0 (0.0) | Inf (0.169-Inf) | 0.471 |
| *NEK1* | 2 (22.2) | 0 (0.0) | Inf (0.169-Inf) | 0.471 |
| *PARP10* | 2 (22.2) | 0 (0.0) | Inf (0.169-Inf) | 0.471 |

Abbreviations: CI, confidence interval; CR, complete response; Inf, infinite; PR, partial response.

*Top 10 genes with lowest *P* values and selected genes of interest from Table 1 are shown.

**Figure S1** Variant selection flowchart for exome data without matched normal samples. Abbreviations: COSMIC, Catalogue of Somatic Mutations in Cancer; ESP, exome sequencing project; ExAC, exome aggregation consortium; dbSNP, single nucleotide polymorphism; FLAGS, frequently mutated genes in public exomes (Shyr C, et al. *BMC* *Medical Genomics* 2014;7:64); GDI, gene damage index (Itan Y, et al. *Proc Natl Acad Sci U S A*. 2015;112(44):13615-20); gnomAD, genome aggregation database; MAF, minor allele frequency; MMY PoN, in-house panel of normals (from the phase 3 multiple myeloma studies CASTOR and POLLUX, Mateos M, et al. *Haematologica* 2020; 105(2):468-477); VAF, variant allele frequency. This figure was published in *Translational Oncology*, Vol 4, Issue 1, January 2021, 100977, Hodkinson BP, et al., *Biomarkers of response to ibrutinib plus nivolumab in relapsed diffuse large B-cell lymphoma, follicular lymphoma, or Richter's transformation,* Copyright Elsevier (2020).


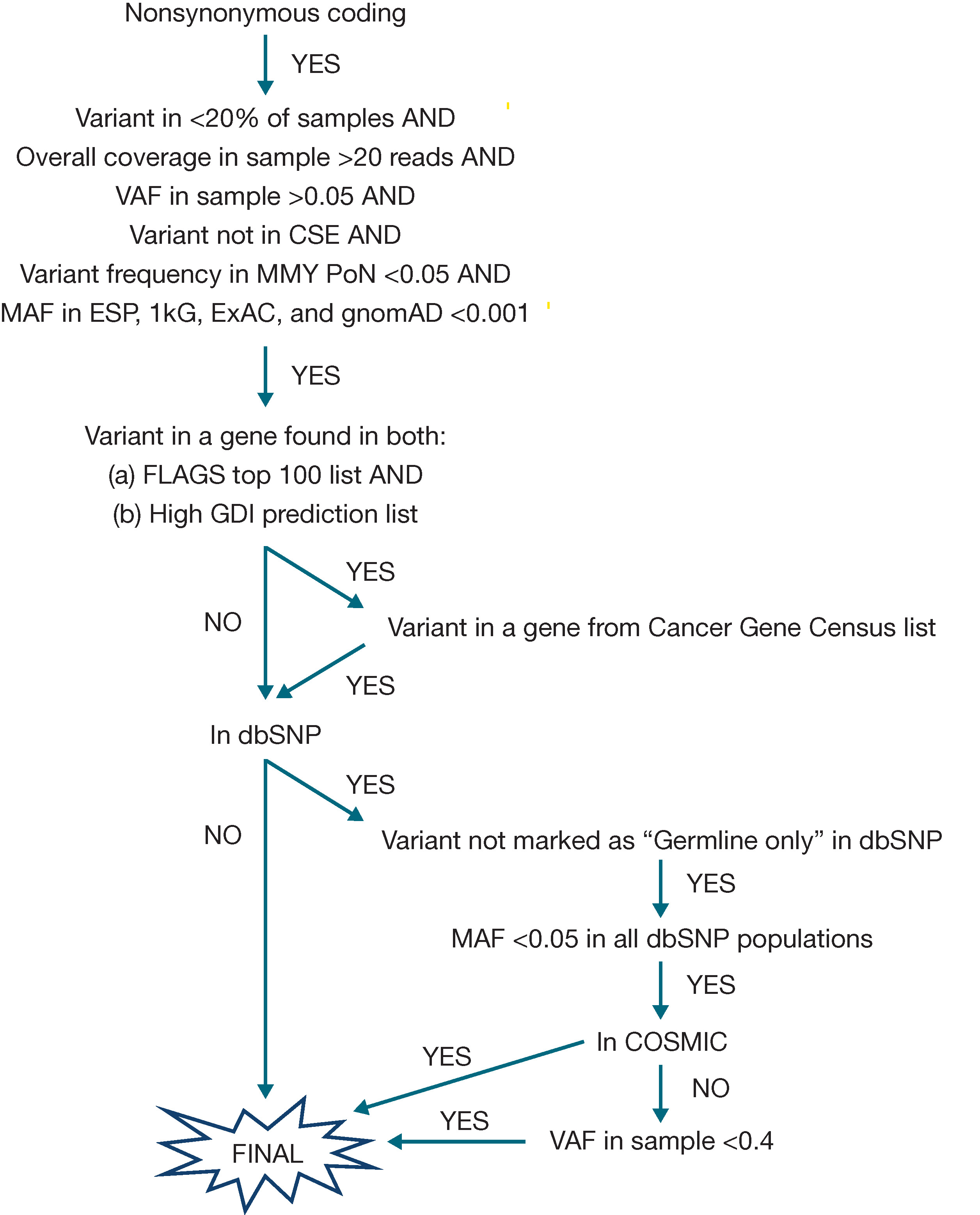


**Figure S2** Histogram for variant allele frequency after in-house reprocessing and variant selection.


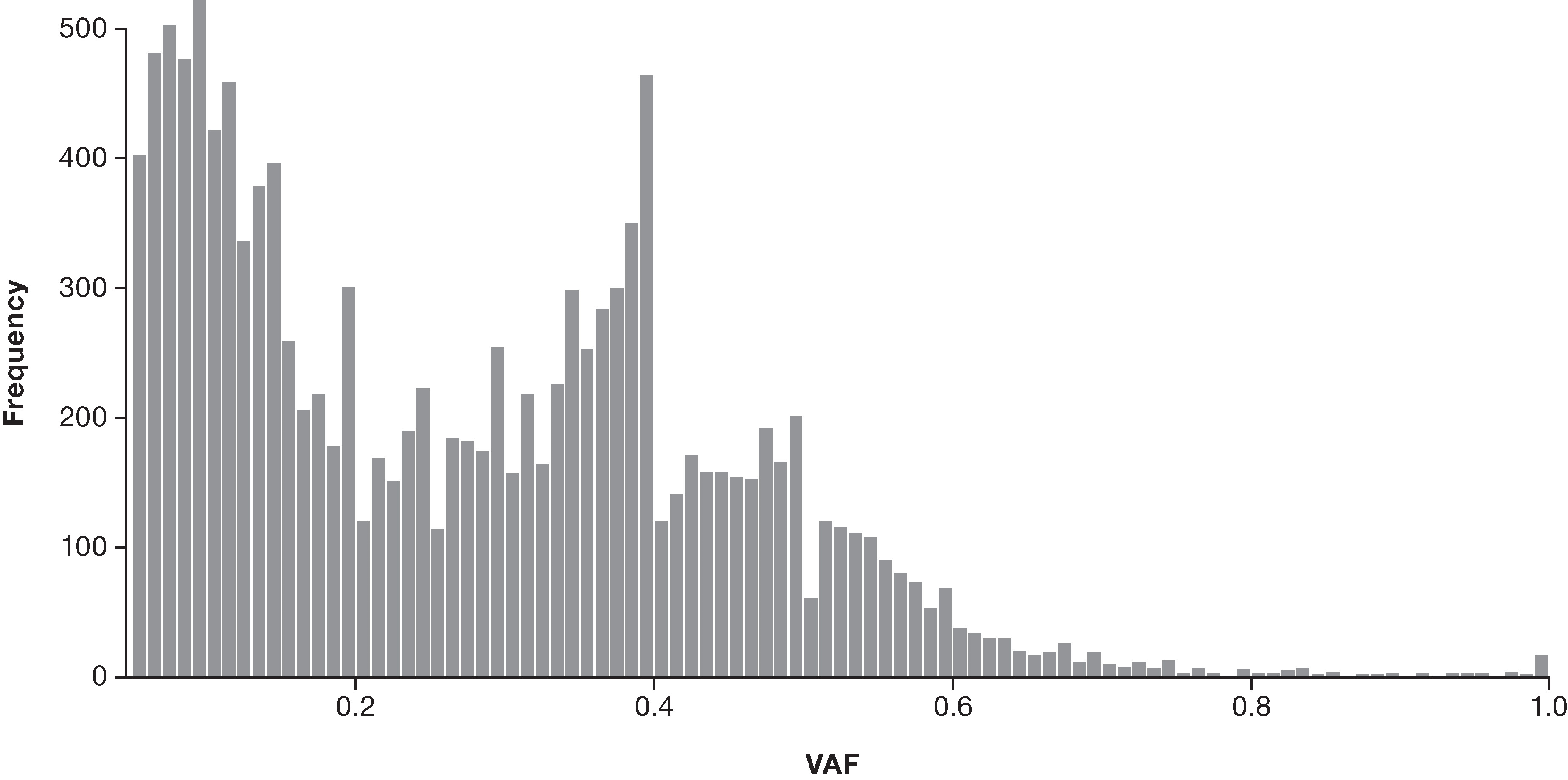


**Figure S3** Number of mutated genes in ibrutinib-treated patients with follicular lymphoma who had response data (n = 83).


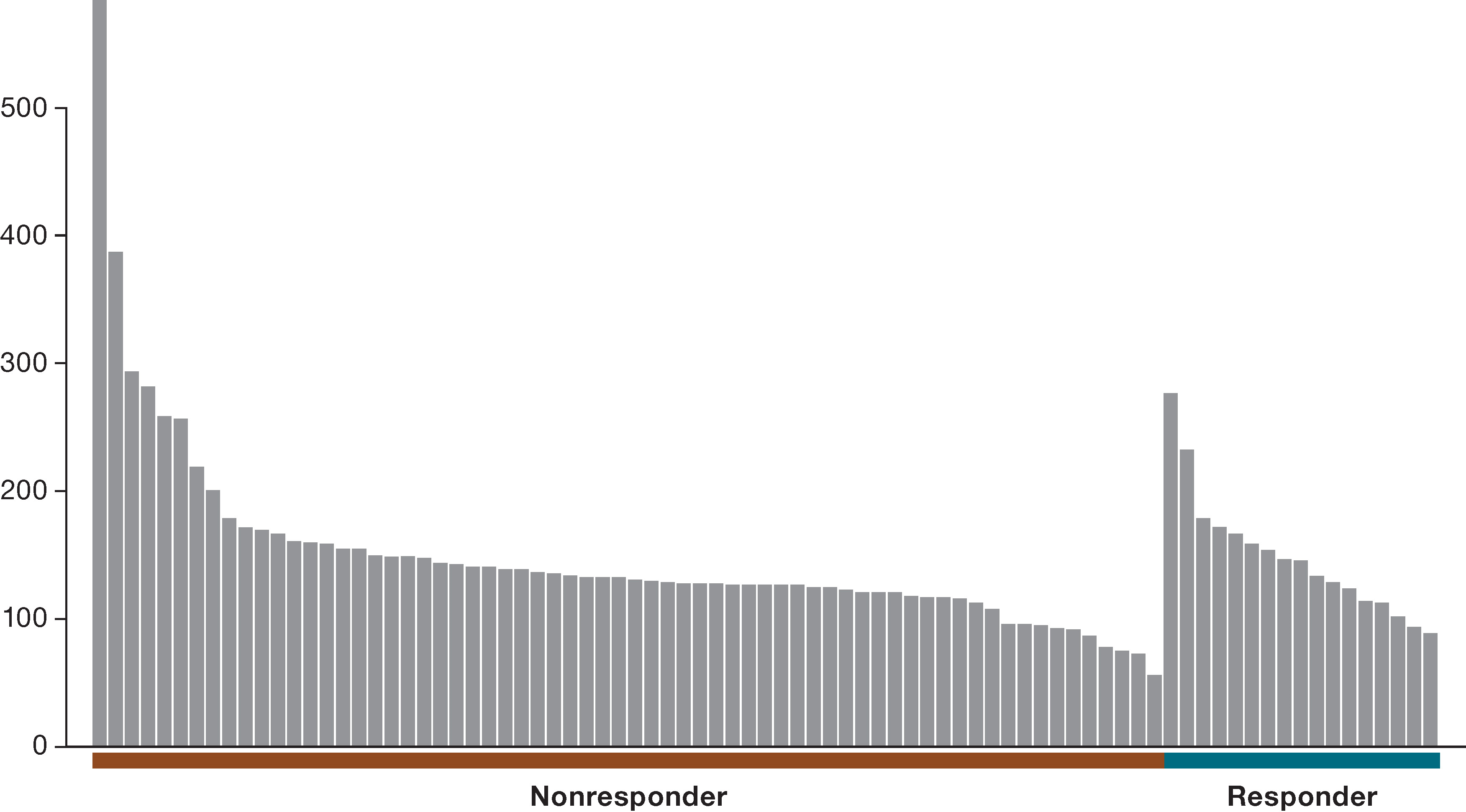


**Figure S4** Heatmap of genes mutated in > 10% of samples (75 genes). Left panel shows the percentage of individuals with a mutation in each gene; right panel shows the distribution of mutations in those genes in the 83 patients for with response/nonresponse data.


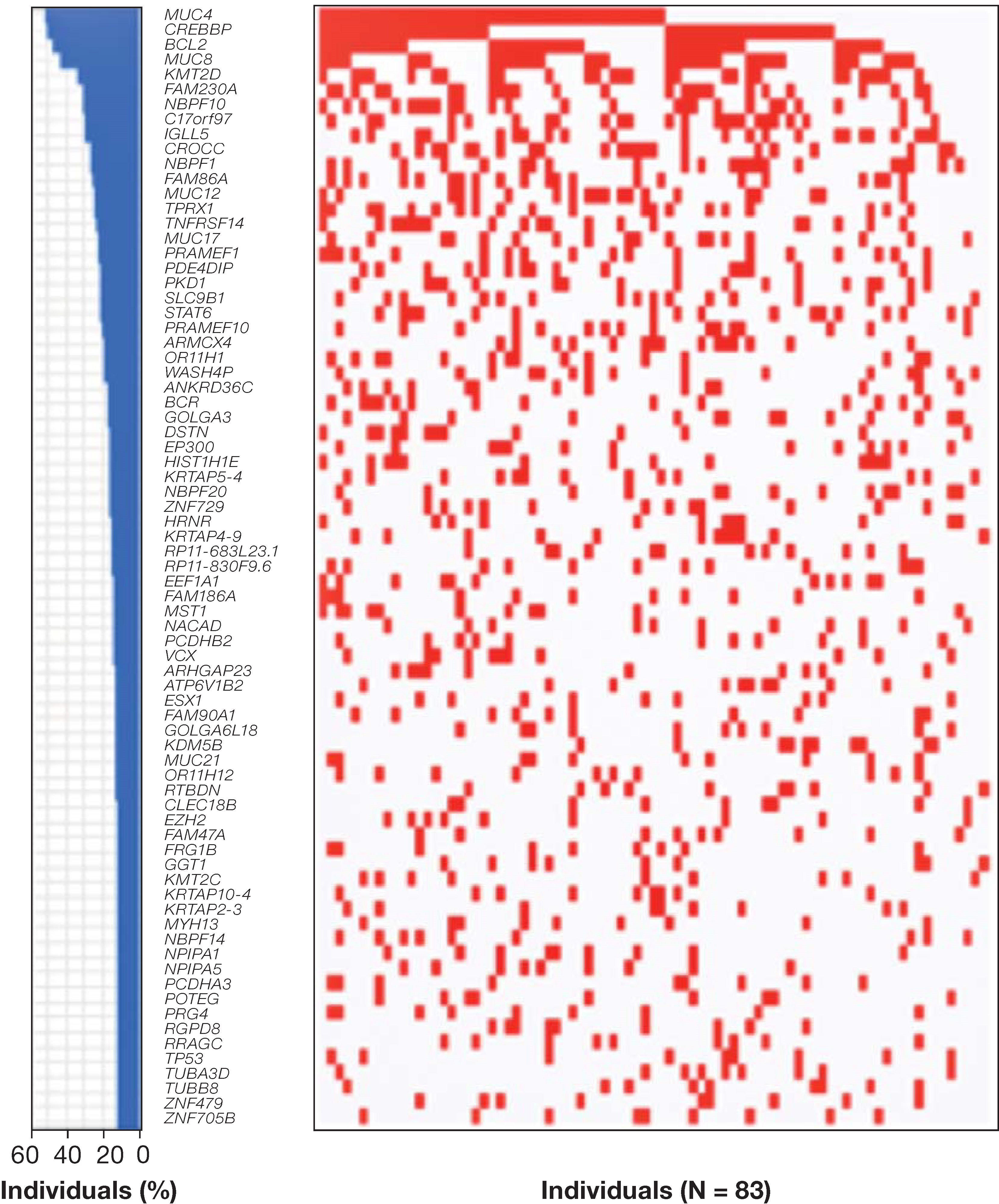

Supplement: Supplementary file 1 — Supplementary Material [file CAM4-11-61-s001.docx]
